# What works for whom in the management of diabetes in people living with dementia: a realist review

**DOI:** 10.1186/s12916-017-0909-2

**Published:** 2017-07-28

**Authors:** Frances Bunn, Claire Goodman, Peter Reece Jones, Bridget Russell, Daksha Trivedi, Alan Sinclair, Antony Bayer, Greta Rait, Jo Rycroft-Malone, Christopher Burton

**Affiliations:** 1 Centre for Research in Primary and Community Care, University of Hertfordshire, College Lane, Hatfield, Hertfordshire, AL10 9AB UK; 2 School of Healthcare Sciences, Bangor University, Bangor, UK; 3 Foundation for Diabetes Research in Older People, Diabetes Frail Ltd, Luton, LU1 3UA UK; 4 Division of Population Medicine, Cardiff University, Cardiff, Wales CF10 3AT, LL57 2EF UK; 5 Research Department of Primary Care and Population Health, UCL Medical School (Royal Free Campus), Rowland Hill Street, London, NW3 2PF UK

**Keywords:** Dementia, Diabetes, Realist review, Realist synthesis, Self-management

## Abstract

**Background:**

Dementia and diabetes mellitus are common long-term conditions and co-exist in a large number of older people. People living with dementia (PLWD) may be less able to manage their diabetes, putting them at increased risk of complications such as hypoglycaemia. The aim of this review was to identify key mechanisms within different interventions that are likely to improve diabetes outcomes in PLWD.

**Methods:**

This is a realist review involving scoping of the literature and stakeholder interviews to develop theoretical explanations of how interventions might work, systematic searches of the evidence to test and develop the theories and their validation with a purposive sample of stakeholders. Twenty-six stakeholders — user/patient representatives, dementia care providers, clinicians specialising in diabetes or dementia and researchers — took part in interviews, and 24 participated in a consensus conference.

**Results:**

We included 89 papers. Ten focused on PLWD and diabetes, and the remainder related to people with either dementia, diabetes or other long-term conditions. We identified six context-mechanism-outcome configurations which provide an explanatory account of how interventions might work to improve the management of diabetes in PLWD. This includes embedding positive attitudes towards PLWD, person-centred approaches to care planning, developing skills to provide tailored and flexible care, regular contact, family engagement and usability of assistive devices. An overarching contingency emerged concerning the synergy between an intervention strategy, the dementia trajectory and social and environmental factors, especially family involvement.

**Conclusions:**

Evidence highlighted the need for personalised care, continuity and family-centred approaches, although there was limited evidence that this happens routinely. This review suggests there is a need for a flexible service model that prioritises quality of life, independence and patient and carer priorities. Future research on the management of diabetes in older people with complex health needs, including those with dementia, needs to look at how organisational structures and workforce development can be better aligned to their needs.

**Trial registration:**

PROSPERO, CRD42015020625. Registered on 18 May 2015.

## Background

Rates of diabetes in people living with dementia (PLWD) are between 13% and 20% [1]. Worldwide there are an estimated 35.6 million PLWD. By 2050 this number will rise to more than 115 million [2]. Although there are differences in the physical and cognitive effects of the different types of dementias, all are progressive, involve increasing physical and mental deterioration and lead to a person with dementia becoming increasingly dependent. In PLWD the likelihood of diabetes-related complications, such as hypoglycaemic episodes, cardiovascular conditions and amputations may be increased [3, 4]. This happens because dementia has an impact on an individual’s ability to administer medication, regulate eating habits and recognise and treat hypoglycaemia [5–7]. Furthermore, there appears to be a reciprocal relationship between hypoglycaemia, dementia and frailty [8, 9]. The impact on health and social care economies and on patients and their families is considerable [10].

The delivery of health and social care for this group is complex and challenging [11]. Despite this, there is currently no systematic approach to the management of diabetes and dementia [12]. In the UK there is a gap in provision of services in mental health trusts for diabetes care and similarly a gap in acute hospital trusts for dementia care [12]. Guidance on the management of diabetes in PLWD outlines a number of recommendations, including better case finding of both conditions, better training for staff, adequate support for family carers and care that is tailored to the need of the individual [12, 13]. However, currently there is little research evaluating interventions to improve the management of diabetes in PLWD; many diabetes-related studies specifically exclude PLWD.

There is a need to consider what kind of interventions are required for the effective management of diabetes in PLWD, including how interventions work, for whom and in what contexts. Interventions designed to improve the management of diabetes in PLWD are likely to be multicomponent, specific to different stages of the dementia trajectory and dependent on the behaviours and choices of those delivering and receiving the care. Realist synthesis is a systematic, theory-driven approach that aims to make explicit the mechanism(s) of how and why complex interventions work (or not) in particular settings or contexts [14–16]. Realist synthesis takes account of a broad and eclectic evidence base, including experiential and clinical knowledge. The aim of this review was to identify programme mechanisms to improve the management of diabetes in PLWD and to identify areas needing further research.

## Methods

### Rationale for using realist approach

Realist review is a theory-driven interpretive approach to evidence synthesis that endeavours to unpack the ‘black box’ of how complex interventions work [14, 15, 17]. The purpose of this review was to develop an explanatory account or programme theory about ‘what works’ in the management of diabetes in PLWD, and in what contexts. Explanatory accounts comprise configurations of context (the background conditions in which interventions are delivered and in which mechanisms are triggered), mechanism (the responses or changes that are brought about through a programme within a particular context) and outcomes. Definitions of key realist terminology used in the review are provided in Box 1. These context-mechanism-outcome (CMO) configurations are developed iteratively through data collection, theorising and stakeholder engagement. This account follows the Realist And Meta-narrative Evidence Syntheses: Evolving Standards (RAMESES) publication standards for realist syntheses [14]. A fuller version of the methods is published elsewhere [18]. In the protocol we specified that we would include people living in the community, a care home or other long-term setting. However, in the scoping phase the inclusion criteria were refined to exclude PLWD in care homes because there were felt to be significant differences between the environments. The change was agreed upon with the Project Advisory Group.

### Phase 1: defining the scope of the realist review — concept mining and theory development

To develop our initial programme theories we scoped published and grey literature (see Table 1) and interviewed 19 content experts. This was followed by a workshop where the project team began to map and prioritise the identified theory. Content experts included clinicians with a special interest in either diabetes or dementia; providers of care in primary or secondary care; user representatives, including recipients of care, family carers and relevant diabetes or dementia charities; and academics and those involved in developing education and guidance for older people with diabetes. Interviews were conducted face to face or via telephone, using an interview schedule guided by realist principles [19]; they were audio recorded and transcribed. Ethical approval was obtained from the University of Hertfordshire Health and Human Sciences Ethics Committee with delegated authority (CHSK/SF/UH/00106).

**Table 1:** Overview of search strategy for Phase 1 and Phase 2 of the review

PHASE 1 SEARCHES	
Phase 1 databases	ProQuest Pro (2010–December 2015): this contains 13 databases including British Nursing Index, PsychINFO, and SocialSciences collection; Cumulative Index to Nursing and Allied Health Literature (CINAHL), MEDLINE, EBSCO, Web of Science, Cochrane Library, Health Technology Assessment (HTA) database, National Institute for Health and Care Excellence (NICE) guidelines, Google Scholar
Focus of search	Search terms (PubMed)
Broad scoping searches	Key words used in the searches included: dementia (Alzheimer’s disease and vascular dementia) diabetes (type 1 diabetes mellitus [T1DM] and type 2 diabetes mellitus [T2DM]), mild cognitive impairment (MCI), frail elderly, self-management (self-care), chronic illness, case-management, assistive technology (telemedicine/care), severe mental illness, family carer, social support, eating/meal times, medicine management (adherence), exercise/leisure, health and social care professionals
PHASE 2 SEARCHES	
Phase 2 databases	1990–March 2016: MEDLINE (PubMed), CINAHL, Scopus, Cochrane Library (incl. the Cochrane Database of Systematic Reviews), Database of Abstracts of Reviews of Effects (DARE), HTA database, National Health Service Economic Evaluation Database (NHS EED), AgeInfo (Centre for Policy on Ageing, UK), Social Care Online, National Institute for Health Research portfolio database, NHS Evidence, Google, Google Scholar
Focus of search	Search terms (PubMed)
A1: Diabetes and Dementia — Theory Area 1 (clinically based approach)	#1 Diabetes search terms: “diabetes” OR “insulin” OR “hypoglycaemia” OR “hyperglycaemia” OR “glycaemic control” OR “glycemic control” OR “hba1c” OR hypoglycaem* OR hypoglycemi* OR hyperglycaem* OR hyperglycem*
	#2 Dementia search terms: “dementia” OR “alzheimer” OR “alzheimers” OR “mild cognitive impairment” OR “cognitive impairment” OR dement* OR alzheimer* OR MCI
	#3 = #1 AND #2
	#4 study type terms: manage* OR treat* OR intervention* OR programme* OR program* OR controlled OR randomized OR randomised OR interview* OR qualitative OR trial OR “randomised controlled trial” OR “intervention study”
	#5 = #3 AND #4
	#6 = #5 Not “cross sectional study” OR “epidemiological studies” OR “case control” OR “cohort study” OR “cross sectional studies”
	All restricted to Title/Abstract, includes both Medical Subject Headings (MeSH) and free text
A2: Diabetes and Dementia — Theory Area 2 (collaborative partnerships)	#1 Same diabetes search terms as A1
	#2 Same dementia search terms as A1
	#3 = #1 AND #2
	#4 Self-management terms: “self care” OR “self management” OR “self medication” OR “self administration” OR “minimally disruptive medicine” OR “adherence” OR “shared decision making” OR “patient preference” OR “patient participation” OR “patient involvement” OR “patient centred care” OR “personalised care” OR “individualised care” OR partnership OR collaboration Filters: Publication date from 1990/01/01
	#5 = #3 AND #4
	All restricted to Title/Abstract, includes both MeSH and free text
A3, B3, C3: Diabetes and Dementia — Theory Area 3 (co-production). This area produced very few hits, so all searches (A, B, C) were combined	#1 Same diabetes terms as A1
	#2 Same dementia terms as A1
	#3 = #1 OR #2
	#4 “co production” OR “co design” OR “codesign” OR “coproduction” OR “co creation” OR co-produc* OR coproduc* OR co-design* OR codesign* OR co-creat* OR cocreat* OR co-commission* OR cocommission Filters: Publication date from 1990/01/01
	#5 = #3 AND #4
B1: Dementia only Theory Area 1 (clinically based approach)	#1 Same dementia terms as A1
	#2 diet OR exercise OR blood glucose OR blood pressure OR medication OR adherence OR self management. Filters: Publication date from 1990/01/01
	#3 = #1 AND #2
	#4 Same study type terms as A1
B2: Dementia only Theory Area 2 (collaborative partnerships)	#1 Same dementia terms as A1
	#2 Same self-management terms as A2
	#3 = #1 AND #2
C1: Diabetes only Theory Area 1 (clinically based approach)	Large area so agreed we would use clinical guidelines (e.g. Sinclair recent guidelines) as these provide up-to-date evidence
C2: Diabetes only Theory Area 2 (collaborative partnerships)	#1 Same diabetes terms as A1
	#2 “frailty” OR “older” OR “elderly” OR “geriatric” OR “elder” OR “aged” Filters: Publication date from 1990/01/01 — all MeSH
	#3 Same self-management terms as A2
	#4 = #1 AND #2 AND #3
Additional search: Tailored Care	#1 Tailored care OR tailoring OR individualised care OR individualized care OR personalised care OR personalized care or needs based care (all MESH)
	**#2** tailored OR tailor* OR individualised OR individualized OR personalised OR personalized OR “needs based” (all Title/Abstract)
	#3 #1 OR #2
	#4 multimorbidity OR multimorbid Or comorbidity OR comorbid OR frailty (all MESH)
	#5 #3 AND #4
	#6 Same study type terms as A1
	#7 = #5 AND #6

Next we developed a series of explanatory accounts containing ‘If-Then’ statements [20]. ‘If-Then’ statements are the identification of an intervention/activity linked to outcome(s), and they contain references to contexts and mechanisms (though these may not be very explicit at this stage). The ‘If-Then’ statements helped to focus the process of considering ideas and assumptions about how interventions work and testing them against the evidence. Initially we generated 20 ‘If-Then’ statements which, after further discussion, were synthesised to three. We then mapped ideas about ‘good’ diabetes care against barriers for PLWD, potential interventions and emerging theory. This became Theory Area (TA) 1 — clinically based approach. Additional theory areas around supportive partnerships (TA 2) and co-production (TA 3) were developed to reflect other areas identified in the scoping.

### Phase 2: retrieval, review and synthesis

In Phase 2 we undertook systematic evidence searches to test and develop the theories identified in Phase 1. The main inclusion criteria were:

- PLWD of any type or severity and type 1 or 2 diabetes, resident in the community
- Any intervention designed to promote the management of diabetes in PLWD and the prevention of potential adverse effects associated with poorly managed diabetes
- Studies that provide evidence on barriers and facilitators to the implementation and uptake of interventions designed to improve the physical health of PLWD
- Studies that offer opportunities for transferable learning such as those that evaluate interventions for PLWD and other clinical conditions, or those that look at the way services are delivered and implemented for PLWD
- Published and unpublished studies of any design, policy documents and guidelines.

The purpose of the searches was not to identify an exhaustive set of studies but rather to reach conceptual saturation [21]. Search terms were devised in conjunction with an information scientist and were chosen to reflect the theory areas identified in Phase 1. The searches were split into three main categories: A — Theory areas + dementia AND diabetes, B — Theory areas + dementia, C — Theory areas + diabetes. More details of the searches are given in Table 1.

### Selection and appraisal of documents

Search results were downloaded into bibliographic software. Records and full-text papers were screened for inclusion by two of four reviewers (FB, PRJ, BR, DT). The reviewers then met to discuss decisions and resolve any disagreements. Decisions on inclusion made at different points in time were recorded in an Excel spreadsheet. Consistent with a realist synthesis approach, items were assessed for inclusion on the basis of whether they were considered ‘good enough and relevant enough’ [22, 23]. This was an iterative process that involved discussion between team members. ‘Good enough’ was based on the quality of evidence, for example whether it was of a sufficient standard for the type of research, and whether the claims made were considered to be trustworthy. ‘Relevance’ related to whether the authors provided sufficient descriptive detail and/or theoretical discussion to contribute to the theories generated in Phase 1.

### Data extraction and synthesis

Data were extracted into a piloted ACCESS database; the included fields reflected the theory areas identified in Phase 1. Data were extracted by one reviewer, with 50% checked by a second. Data in a realist sense are not just restricted to the study results or outcomes measured but also include author explanations and discussions, which can provide a rich source of ‘data’ that makes explicit how an intervention was thought to work (or not). The ACCESS database was used to identify prominent recurrent patterns of contexts and outcomes (demi-regularities) in the data and the possible means (mechanisms) by which they occurred [24]. This deliberative and iterative process enabled iteration from plausible hypotheses to the uncovering of potential context-mechanism-outcome (CMO) configurations. The data were discussed with the entire project team at a second workshop.

### Phases 3 and 4: test and refine programme theories (validation) and develop actionable recommendations

To develop a final review narrative we reviewed the hypotheses through consultation with the Project Advisory Group and with stakeholders. Stakeholder consultation was done via telephone interviews (*n* = 7) and group discussions at a consensus conference (*n* = 24). Participants at the conference were purposively sampled to ensure that all the stakeholder groups in Phase 1 were represented. Following the consensus conference the CMOs were revised and rechecked against data from the literature and stakeholder transcripts.

## Results

**Fig. 1:**
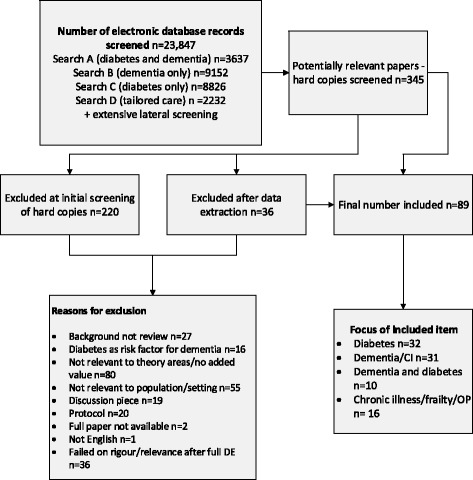
Overview of study selection process

We included 89 papers [8, 11, 12, 25–110]. These included 79 research papers (22 reviews, 57 primary research papers) and 10 guidelines or discussion pieces. The 57 primary research papers reported 51 studies. Ten papers focused on people living with dementia and diabetes (PLWDD), and the rest were concerned with diabetes (*n* = 32), dementia (*n* = 31) or other groups, such as those with chronic illness or frailty. An overview of the selection process is provided in Fig. 1 and a summary of included studies in Table 2. The majority of the studies we included were from the UK (*n* = 33), North America (*n* = 23) or mainland Europe (*n* = 11).

**Table 2:** Overview of included studies

Focus	Methodological approach	Types of outcomes
Diabetes AND dementia (*n* = 10) • Clinical guidance on management of diabetes in PLWD • Impact of dementia on self-management (SM) • SM support for people with cognitive impairment (CI)	• 3 literature reviews • 1 controlled (not randomised) study • 1 guideline • 2 qualitative • 1 description of a service • 1 cross-sectional survey • 1 mixed methods	• Glycated haemoglobin (HbA1c) • Hypoglycaemia • Impact of dementia on SM Note that only one study evaluated an intervention (Camp 2015)
Dementia NOT diabetes (*n* = 31) Includes: • SM support for PLWD • Support for family carers • Medication management • Home-based support for PLWD (e.g. delivered by an occupational therapist) • Cognitive rehabilitation	Study type • 9 qualitative • 9 systematic reviews • 7 randomised controlled trials (RCTs, 3 papers report 1 study) • 6 other	Patient outcomes include: • Cognitive function • SM-related behaviours such as knowledge and self-efficacy (but not diabetes focused) • Quality of life • Experiences and views • Mood Carer outcomes include: • Carer stress, burden, quality of life • Experiences and views
Diabetes NOT dementia (*n* = 32) Participants include older adults, those with complex health needs (comorbidity, frailty, etc.), people with mental illness and adults with type 2 diabetes mellitus Includes: • Interventions related to improving SM (e.g. use of assistive technology (AT), SM support or decision aids) • Better understanding the needs of people with diabetes	• 5 guidelines • 9 other (variety of study designs) • 7 qualitative • 5 RCTs • 5 systematic reviews	• Glycaemic control • Patient knowledge, self-efficacy • Diabetes self-care practices • Feasibility and acceptability of interventions • Quality of life • Views and experiences
Other (e.g. people with chronic illness, frail older people, people with multimorbidity or long-term condition) *n* = 15 Includes: • SM support for conditions other than diabetes • Service organisation • Use of AT in older people	• 5 qualitative • 4 RCTs • 1 systematic review • 4 other	• Views and experiences • Self-care related outcomes (e.g. knowledge, self-efficacy) • Use of AT

Our review resulted in six CMO configurations which provide an explanatory account of how interventions might work to improve the management of diabetes in PLWD (see Table 3). Supporting evidence from stakeholder interviews is provided in Table 4.

**Table 3:** The six context-mechanism-outcome configurations and supporting citations

Title	Context	Mechanism and outcome	Included evidence
1. Embedding positive attitudes towards PLWD	If health and social care delivery systems propagate and reinforce positive attitudes towards people living with dementia and diabetes (PLWDD) and their families, through tailored self-management support	Then this fosters a belief in staff that PLWDD have the potential to be involved in self-management (SM) and the right to access diabetes-related services (even when the trajectory is one of deterioration) (M) prompting treatment confidence in PLWDD (M), which leads to engagement in SM practices by PLWDD and their carers (O)	[11, 12, 28, 37, 39–41, 53–55, 62, 67–70, 73, 74, 80, 81, 88, 99, 100, 102, 104, 110]
2. Person-centred approaches to care planning	If delivery systems promote a person-centred and partnership approach to care, allowing healthcare professionals (HCPs) to understand the individual needs and abilities of PLWDD and their family	Then (1) HCPs feel confident that they are acting in the best interests of PLWDD and family (M), and this (2) generates trust between HCP and PLWDD/family (M), leading to better fit between care planning and patient and carer needs and (potentially) a lessening of the burden of medicalisation experienced by PLWDD and their families (O)	[26, 27, 31, 35, 37, 42, 43, 52–54, 58, 60, 62, 64, 65, 72, 75, 79, 82, 84, 89, 90, 93–95, 98, 100, 102, 105, 106]
3. Developing skills to provide tailored and flexible care	If HCPs are expected to develop skills that enhance the delivery of individualised and tailored care to PLWDD (e.g. enablement rather than management, listening/communication/negotiation)	Then this legitimates the work creating the expectation in patients and HCPs that the management of diabetes for PLWD is important (M), leading to the provision of more tailored diabetes care (O) and better engagement in self-management by PLWDD and family carers (O)	[8, 26, 35, 44, 45, 58, 62, 64, 75, 78, 79, 82, 83, 85, 89, 91, 97, 102]
4. Regular contact	If HCPs maintain regular contact over time (e.g. face-to-face, telephone, e-mail) with the PLWDD/family, monitoring and anticipating needs throughout the dementia trajectory	Then HCPs feel more equipped to meet patient needs (M), and PLWDD/family believe themselves to be supported (M) through transition from functional independence to functional dependence (M), leading to improved diabetes management (O)	[12, 26, 34, 36–38, 41, 43, 46, 57, 82–86, 92, 96, 98]
5. Family engagement	If family carers are routinely involved in care planning and information sharing and are given the support they need to take on the tasks associated with managing diabetes in PLWD (e.g. medication management, recognition of hypoglycaemia)	Then family carers will feel supported and believe their contribution is recognised and appreciated (M), leading to the development of effective self-management strategies on the part of the family carers (O)	[11, 25, 26, 31, 33, 46, 47, 53, 64, 71, 72, 81, 92, 98, 110]
6. Usability of assistive devices	As the dementia trajectory progresses, assistive technology needs to be tailored and adapted to the needs and requirements of PLWDD and family (includes social, environmental and cultural needs) with the focus on maintaining autonomy for the PLWDD	This leads to PLWDD and family gaining awareness of the usefulness of assistive technology in their management of diabetes and dementia (M), leading to more effective and sustained use of assistive technology to maintain autonomy and diabetes self-management strategies (O)	[11, 25, 34, 37, 48, 49, 51, 56, 59, 63, 69, 76, 77, 87, 95, 101, 103]

**Table 4:** Examples of supporting evidence from stakeholder interviews

CMO	Examples of supporting evidence from stakeholder interviews
1. Embedding positive attitudes towards PLWD	• “…you shouldn’t be sort of swayed one way or the other, just because someone has dementia… I think certainly when they first start on their journey I think it’s really important that we do everything we can…” [of cross-disciplinary training to facilitate appropriate care], Diab1
	• “…an intervention should work at a level that people…particularly early stages of dementia…can be included…so it’s not decisions being made about them…”, Dem1
	• “…the Getting To Know Me project here in XXX we trained over six hundred, or seven hundred, frontline practitioners about dementia, just a general aspects of dementia, what it is, how to communicate, what to look for, what people might be saying when they maybe can’t tell you through words…”, Dem7
2. Person-centred approaches to care planning	• “But actually at this stage (referring to when people have complex health needs) people are interested in autonomy, mobility you know, retaining as much function and independence as they can, being a burden on their families you know, so all the normal things and they’re often much, much more important than a lot of the medical stuff”, Diab12
	• “It’s allowing a two-way exchange of information isn’t it about how different conditions might affect things.”, Res1
	• “Where the client is fully able to contribute [to a care plan], that usually goes very well because we can then discuss the likes and dislikes, their routines, how they manage their diabetes themselves…”, Dem4
	• “I think different targets for certain groups of people you know, and quality of life targets rather than all about number crunching…”, Diab13
3. Developing skills to provide tailored and flexible care	• “…we encourage people to set agreed targets with the patient…that may well be…higher than the general population target which is a key message we get across to the GPs because they’re so driven by QOF”, Diab9
	• “I don’t think we’re supporting people with diabetes [and dementia] as well as we could, because of this training issue and where responsibility lies…”, Dem4
	• “I’ve seen very very few examples where it’s done well, any of this, any of this sort of self-management, shared decision-making, anything. …..I think health professionals are possibly becoming themselves much more risk-averse and not wanting to suggest things that aren’t perceived as being healthy or might not be the right answer”, Res1
	• “choosing wisely American stuff you know, I think we’re all warming up to this agenda but I don’t think anyone’s quite cracked you know, it’s not mainstream yet.”, Diab12
	• “…for the general population, self-management…is not working particularly effectively…translate that to a much more delicate and fragile group…who have other comorbidities and have dementia…then those types of responses are likely to be even less effective …” [of SM support strategies], Diab2
	• “…one of my profound frustrations is that you can have people talking about multiple long-term conditions and they’re excluding mental health diagnoses, and yet we know that you know, 30% to 40% of people with diabetes will have anxiety and depression you know, and often early unrecognised memory issues…”, Diab12
4. Planned regular contact	• “…if it’s set up on a regular basis, so the person knew, you know, like Tuesday afternoon’s when I speak to my diabetic nurse, that can be put in their diary.”, Dem4
	• “since I’ve been in the care of the Diabetic Clinic everything else has gone out the window. When I was in the care of the specialist nurse at the GP’s, I would have a regular sort of every six month check on my feet…and the amount of protein in my urine, all those tests have now ceased, I’m now only looked at from a point of view of sugar levels.”, Person with type 2 diabetes mellitus
	• “…if you have one healthcare person who you know is almost like your keyworker, your key contact, you build up a relationship, which is very important…”, Diab4
	• “…I have a very good colleague…who is a specialist physical healthcare nurse and a mental health nurse, and that is a, I’ve often thought of this person as a really interesting model for the future…”, Dem7
5. Engaging with families	• “…I think we could probably do a lot more…supporting families and carers and to give them the confidence, I think they’re so worried, it can be so… frightening…to have both conditions…”, Diab13
	• “… patients are educated one to one or through diabetes-structured education, again I’ve never heard of a patient education for carers and those with dementia to support them…”, Diab11
	• “We need to sort of normalise the situation where it is completely normal and expected that close family members will be involved in any decisions and there will be partnership”, Researcher, self-management of long-term conditions
6. Usability of assistive technology	• “… there’s electronic dosette boxes…linked to telecare, so if the person doesn’t take the medication, telecare will come through the intercom and say, ‘Mr so and so, you need to take your tablets’, and then if they don’t …[it] locks anyway so they can’t overdose”, Dem2
	• “I was familiar with the sort of dosette box, if that’s what you’re talking about, which is a great idea and does really help people”, Dem1
	• “But, I mean some of the insulin pens are really fiddly as well, like they’ve got really tiny numbers and you have to dial it up and all that kind of thing, I don’t know how well they’re adapted for people with visual problems or cognitive problems.”, Dem6
	• “In one local authority, we went to one recently, a dementia kind of carers group and sat with them and showed them a list from somewhere else and some of the devices on that weren’t on theirs, you know? It’s not equitable…”, Res2
	• “…technologies are great but you need to think is it the right thing for the patient…is there enough support around it to implement it and respond to it …”, Res2

### CMO 1: embedding positive attitudes towards PLWD

PLWD face problems accessing healthcare. This includes a failure to design services around their needs, poor communication between services, a lack of training on dementia care for health and social care staff, and a reliance on others (such as family carers) to recognise a need for services and stigma [11].

### Involving PLWD in self-management

Ten studies looked at self-management (SM) interventions for PLWD or cognitive impairment (CI) [37, 41, 70, 73, 74, 80, 81, 88, 100, 104]. The evidence is limited, as most focus on people living with mild or early stage dementia, are mostly qualitative or small pilot or feasibility studies, do not report measurable health outcomes and usually focus on people with a partner [111]. Furthermore, only one study, a controlled evaluation of personalised education sessions, included people with both dementia and diabetes [37]. This US-based study found a significant increase in self-efficacy but no difference in glycated haemoglobin (HbA1c) levels at a 6 months follow-up.

**Fig. 2:**
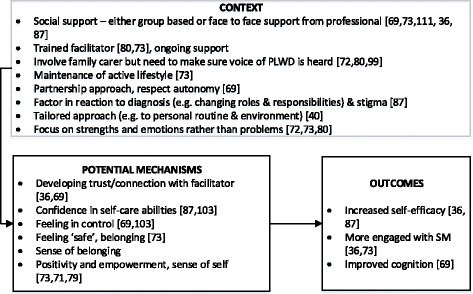
Overview of identified context, mechanisms and outcomes for self-management programmes in PLWD

Despite this, these studies offer evidence and transferrable learning for the development of SM support for PLWDD. For example, confidence and a sense of control appeared to be important mechanisms that could lead to increased engagement in SM activities for PLWD [98]. Several studies suggested that supporting service users and carers to become more functional, independent and resilient was preferable to a purely clinical focus on managing or treating medical symptoms [42, 54, 106]. Work on enablement suggests that there is a link between independence, functional ability and self-care behaviour, and feelings of confidence or self-efficacy in PLWD and their family carers [39, 41, 98]. Working with families is clearly key. However, studies highlight the need to ensure that the voice of the PLWD is heard [81]. Qualitative studies on SM support for PLWD found that information provision may be aimed at carers, leaving PLWD feeling powerless [81], and that PLWD can find support inappropriate or stifling [73, 100]. An overview of context, mechanisms and outcomes for promoting SM in PLWD is shown in Fig. 2.

### CMO 2: person-centred approaches to care planning

PLWDD have two chronic life-limiting conditions with different trajectories. Dementia generally has a progressive or stepwise pattern of progression, whereas diabetes may have a more constant course with longer periods in which to adapt [112], but the trajectory of each is likely to have an impact on the other. Delivering appropriate and sustainable care for PLWDD from early stage to advanced dementia is a difficult clinical enterprise that requires a change from a curative, biomedical strategy to a more person-centred approach where patient priorities are at the forefront [102, 113].

A recognition of patient motivators and goals and negotiation of a mutually agreed management plan could improve adherence to SM regimens [26, 58, 65, 84, 93]. For example, maintaining independence and engagement with day-to-day activities was a clear priority for participants in all groups (e.g. PLWDD, older people with diabetes, PLWD) [32, 37, 60, 78, 79, 100]. Several studies suggested, however, that there was ‘goal divergence’ between patients, carers and HCPs, particularly when patient care was complicated by the presence of multiple conditions [32, 58, 102].

Whilst continuity of care in long-term conditions such as diabetes is known to be important, it assumes an added significance for the PLWDD and their family. A long-term trusting relationship is a key element to achieving a person-centred approach to care that prioritises and values the patient’s subjective health experiences [11, 26, 27, 31, 32, 43, 64, 65, 84, 93, 110]. People with diabetes and CI are likely to need long-term connection and maintenance programmes in order to maintain positive impacts on glycaemic control [37].

Patient-centred communication and collaboration between healthcare professionals (HCPs) and patients/families appear to be key to achieving individualised care [32, 58, 89, 102]. Several studies looked at the use of tools, such as decision aids, for involving older people with diabetes in shared decision making [35, 75, 89]. These did not show an impact on glycaemic control, but the tools were thought to facilitate and provide structure for patient-centred practice, co-construction of decision making and patient engagement in their diabetes management. However, whilst such tools may be useful, PLWDD are likely to need interventions which involve more frequent contact and include repetition and reinforcement. Decision making is likely to be complicated by issues around consent, concordance and the appropriateness of treatment.

### CMO 3: developing skills to provide tailored and flexible care

The literature highlights the importance of assessing the specific self-care barriers faced by older people with diabetes, and tailoring advice and support to individual needs and goals for diabetes SM [82, 94]. Clinical guidelines on diabetes recommend that target HbA1c levels be relaxed for older people who are frail or have comorbidities and/or dementia [12, 114–116]. For example, a Best Clinical Practice statement recommends that, for PLWD, clinicians should aim to achieve a fasting blood glucose 6–9 mmol/L, range (HbA1c 53–64 mmol/mol; 7–8%) [12]. Despite this, many older people with diabetes are potentially overtreated [8]. This could happen partly because these targets do not ‘fit’ with current performance measures [117] (such as Quality and Outcomes Framework (QOF) targets in primary care in the UK), or because HCPs do not always have the skills needed to provide flexible individualised care for people with diabetes and/or dementia [11]. Moreover, because SM support does not fit with a biomedically focused ethos, it is not embedded in the day-to-day work of primary care [60, 66–68, 85, 97].

A case study involving the use of continuous glucose monitoring to measure hypoglycaemia in older adults with diabetes found that simply relaxing HbA1c goals may not be adequate to protect frail older adults against hypoglycaemia. The authors concluded that there is a need for treatment regimens that better match patients’ self-care abilities [83]. However, research suggests that many HCPs do not take into account the abilities of patients and their family carers to cope with prescribed treatment [32, 82].

### CMO 4: regular planned contact

Planned, regular contact between HCPs and the PLWD carers is needed in order for HCPs to anticipate transitions and help PLWDD and their family carers to manage changes in function and SM capabilities [110]. This is particularly important for PLWD where the dementia may progress in an uneven pattern of decline [12, 36, 50] and where the transition from autonomy to delegation or to caregiver-led management may be particularly difficult [11, 26, 84]. In a trial focused on improving diabetes management in older people, the authors found that older adults were reluctant to make changes to medication between clinic visits. However, regular phone contact from a diabetes educator encouraged people to adjust insulin dosage, leading to better glycaemic control [82].

The studies in this review describe a range of interventions delivered by a variety of HCPs: most commonly general practitioners (GPs), nurses, occupational therapists, psychologists and — in US studies — Certified Diabetes Educators (CDEs). Regardless of who is delivering the intervention, studies consistently highlight the importance of continuity and the quality of the patient-clinician relationship [11, 37, 41, 46, 57, 82, 98]. Whilst the quality of the relationship is important, PLWDD need to be managed by practitioners with appropriate expertise. Dementia as a comorbidity may challenge a diabetes specialist, and a dementia specialist may lack appropriate diabetes knowledge. Ensuring that all professionals have expertise in diabetes and dementia would be difficult, and a collaborative practice is likely to be necessary for people with both conditions, particularly for more complex cases such as people who are insulin-dependent or those with advanced dementia [91]. The way in which specialists involved in the long-term care of patients with dementia and diabetes or other long-term conditions liaise with each other was recognised as an issue, but was outside the remit of this review.

### CMO 5: family engagement

There is a great deal of evidence that family members often provide significant SM support for people with long-term conditions such as diabetes [26, 64, 90], particularly when dementia affects a person’s ability to undertake self-care-related tasks [110]. Family members are also often proactive in facilitating continuity, negotiating access to services for their relatives [11, 110] and providing support or motivation for their relatives’ SM activities [25]. Despite this, family carers often feel undervalued or excluded from decision making, and they may be ill prepared to take on responsibility for SM [11, 26, 46, 47]. Problematic medication management practices may persist despite the involvement of a family carer [26, 71]. The situation is often further complicated by the fact that carers may only take on SM-related tasks once there is a crisis or a failure to adhere to medication [47].

Managing the needs of a family member with diabetes and dementia raises particular anxieties for carers because of the risk of hypoglycaemia and other adverse events associated with diabetes. Ensuring that their family member eats appropriately and that this is coordinated with their medication is a source of great concern for carers, particularly if they are not co-located [11]. Inflexible service provision, such as district nurses being able to go in only at certain times of the day or social services carers not having the authorisation to oversee medication for people with diabetes, is an additional concern [11, 110].

Although we found no studies evaluating structured interventions to provide education and support to family carers of PLWD, qualitative studies looking at SM for PLWD [81] and for people with diabetes (not dementia) [64] argue that it is important to involve carers in the development of SM skills alongside the person they care for. Interventions aimed at supporting PLWD to manage their diabetes should take into account the education and support needs of family carers as well as the PLWD [11, 26, 46]; this support needs to include the issues arising from both conditions and the way dementia is likely to affect diabetes management [11].

### CMO 6: usability of assistive technology

For the purpose of this CMO, assistive technology (AT) is defined as ‘any product or service designed to enable independence for disabled and older people’ [118]. We included 17 papers (five of which were systematic reviews) in this section. Two relate to PLWDD [11, 37], seven relate to the use of AT in PLWD [34, 48, 49, 51, 63, 69, 95], four concern the use of AT in older people [56, 87, 101, 103] and four involve people with diabetes (not dementia) [25, 59, 76, 77]. Current evidence suggests that AT may facilitate communication and access to support and information for carers, but that it has little impact on independence, safety or security for PLWD. There are also issues with the performance of the technology and acceptability to users [48].

AT such as biometric monitoring, medicine management reminders and sensors and alarms to track movement is seen as one way of maintaining autonomy for PLWD [103]. The use of simple technology such as dosette boxes can also be used to maintain independence, although these tools are likely to become less successful as the dementia progresses [110]. AT provision often involves the direct purchase of ‘off-the-shelf’ technology which is adapted by family carers [49, 56]. A review of AT for PLWD found that family carers were most likely to use technology not specific to caring or to dementia, such as tablets, baby monitors, smart phones or light sensors [69].

Family carers played a significant role in supporting the use of AT [48, 49, 76, 77, 87, 101, 110]. They helped to embed AT, reduce patient anxiety [49] and increase motivation and participation [25]. However, a feasibility study of diabetes self-care support suggests that family ties are complex and that family involvement in SM is not always helpful [77]. There is evidence that PLWD wanted to and could contribute to the design of AT, and commentators argue that this makes it more likely to ‘fit’ with the needs of the PLWD [95]. However, even when they are involved in the design of AT, PLWD are likely to need on-going support and family involvement [101]. Technology in itself is unlikely to solve the problem of independent living for older people [56], particularly for those living with dementia [69, 119]. Technology appears to be most effective when it augments or involves face-to-face contact [34, 37, 48, 59].

### Summary of CMOs

**Fig. 3:**
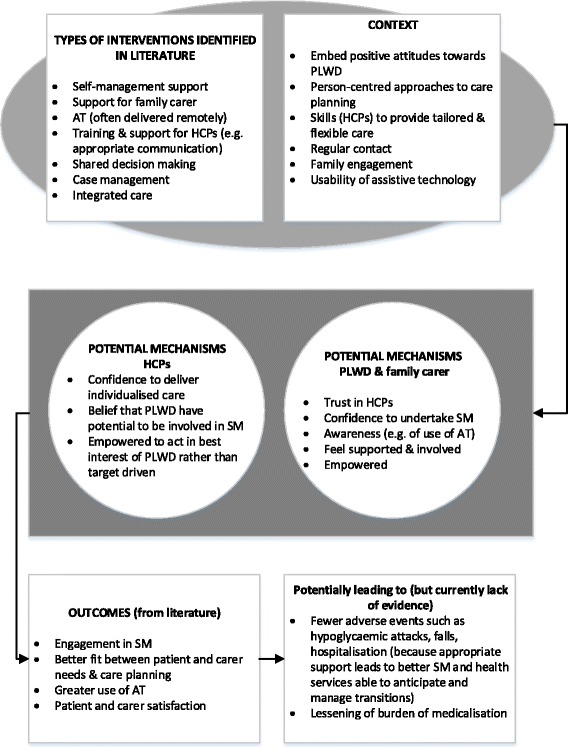
Summary of the six CMO configurations that make up the programme theory

The six CMO configurations are summarised in Fig. 3. The CMOs are grounded in evidence from the literature and stakeholder perspectives. Although designed to be specific to PLWD, the CMOs are also likely to be transferable to other groups who experience problems with diabetes management, for example older people with complex health and social care needs. The CMOs are not mutually exclusive, and we would suggest that what is most important is how the different elements of each interact. The CMO configurations require changes in individual or organisational behaviour or understanding, and in many cases both. For example, CMO 3 — which focuses on skills development — requires the development of skills at an individual level but also organisational changes which legitimise the importance of those skills and allow the time for them to be acquired and practiced. The outcomes we specified in the protocol for this synthesis included a number of clinical outcomes, such as the prevention of hypoglycaemia, the management of cardiovascular risk factors and the identification and management of long-term complications such as neuropathy. However, the outcomes that emerged from the evidence available are primarily experiential rather than clinical, focusing on the need to trigger mechanisms such as trust, confidence and empowerment.

## Discussion

### Summary of findings

An overarching contingency emerges from the data that relates to the convergence or alignment between an intervention strategy, disease progression and social and environmental factors, in particular the involvement of family carers. In the early stages, where the PLWDD can still retain some functionality to make decisions about their diabetes management, ‘personalisation’ and ‘relationship’ building are key components of care that involve the PLWDD, their family carer and the HCPs. This requires a re-orientation of staff capabilities towards prioritising PLWDD and family perspectives over biomedical directives. As the dementia trajectory progresses and independent functioning becomes more problematic for the PLWDD, affecting SM behaviours, then there may be a need to move towards greater ‘monitoring’ by the HCP and family carer. This risk-management perspective may use technology as a way of maintaining confidence in diabetes SM, but we found no evidence to suggest that it can compensate for the loss of cognitive ability. The relationship and personalisation perspectives are essential throughout the trajectory, but they are adapted to anticipate the changing needs of PLWDD and their family carers.

### Implications of the findings

This review highlights the way that emotional support and practical assistance provided by families is key for PLWDD. Despite this, carers often feel undervalued or ill prepared to take on caring responsibilities. A survey of carers of older people with diabetes found that 40% of family carers had never received any information on diabetes from professionals [120]. Many of the support needs of family carers identified could equally apply to PLWD and other comorbidities. However, there are clearly some concerns that are particularly pertinent to family members of PLWDD. Whilst many carers may find medication management stressful, this task is often exacerbated for family carers of PLWDD owing to their concerns about the prevention of adverse events associated with either hypoglycaemia or hyperglycaemia [11, 46, 110]. Family carers are likely to need education combined with on-going support from a specialist in diabetes [12]. They may also have needs related to the dementia, such as how to manage behaviours that challenge.

There is little evidence on which to base any recommendations for practice for PLWDD who have very limited networks of support from family or friends. Research suggests that PLWD who live alone have an increased risk for unmet social, environmental, psychological and medical needs [121] [11]. Our CMO on person-centred approaches to care planning identified the generation of trust between the HCP and the PLWD as necessary in order to improve care planning and SM. Building trusting relationships with HCPs may be particularly important for those who live alone, as they do not have a family member to facilitate access and continuity of care [11] and are likely to be more dependent on HCPs to perform this role [122]. However, PLWD who lived alone found it difficult to trust others and admit to their mistakes or challenges, because they feared being placed in long-term care [123].

Person-centred care is a consistent feature of our programme theory. It is identified as a trigger for mechanisms such as trust, empowerment and a belief that SM is achievable and worthwhile for people with dementia and diabetes. These ideas are not new, as guidance for both conditions recommends person-centred care [61, 124, 125]. What this synthesis does is begin to identify some of the key components of person-centred diabetes care for people with dementia and the organisational and practice changes this might entail. For example, the instigation of individualised (possibly simplified) diabetes regimens requires that the PLWDD sees an appropriate specialist/s or that generalists such as GPs and practice nurses have appropriate knowledge about how to tailor diabetes care. It presupposes interprofessional approaches to clinical decisions and regular review of care. It also relies on HCPs knowing that such actions are legitimised, for example through less focus on biomedical targets and time allocation to establish an understanding of the individual’s story and priorities. This is inevitably linked to continuity and having a practitioner who both understands the dementia trajectory and can also respond as care needs alter [11].

A UK study found that PLWD had an average of 4.6 chronic diseases in addition to their dementia [126], meaning that diabetes may be only one of several healthcare concerns for older people with dementia. In addition, diabetes and frailty are closely interrelated [127], and the relationships between hypoglycaemia, frailty and dementia appear to be reciprocal, with each condition potentially exacerbating the others [8]. Guidelines on diabetes care in older people and those with dementia emphasise the need to consider the significance of frailty and the need to avoid hypoglycaemia [12, 114]. Despite this, there is evidence that a substantial proportion of older adults are potentially overtreated [128]. Performance measures should incentivise appropriate de-intensification as well as intensification of medication regimens [8], and research is needed to consider whether care pathways for this group need to be specific to diabetes and dementia or whether a pathway for older adults with complex needs, such as frailty or multimorbidity, is more appropriate.

### Strengths and limitations

The main limitation of this study was the lack of evidence relating specifically to the management of diabetes in PLWD. This lack of evidence is compounded by few insights into how the point at which the person develops dementia or diabetes affects treatment. However, in realist methodology the unit of analysis is the programme theory, or underpinning mechanism of action, rather than the intervention [129]; as such we were able to draw on a wider body of literature that provided opportunities for transferable learning. This enabled us to develop a theory-driven explanation in the form of six CMO configurations that can be used to guide future initiatives and interventions.

The outcomes in our CMOs are largely experiential rather than clinical. This reflects the evidence available. Outcomes such as increased engagement in SM are potential surrogates for better clinical management of diabetes, but this is not proven. The literature suggests that key goals for this group are maintaining independence and creating treatment regimens that ‘fit’ with the needs and abilities of the PLWD and family carer. However, literature in this area is scarce, and further work is needed to identify what it is that PLWDD and their family carers want from interventions [130].

Much of the evidence we included related to either PLWD or people with diabetes, rather than people with both conditions. Inevitably, the aims, focus and outcomes of these two sets of studies are very different. Moreover, because we drew on this larger literature, there were many more potentially relevant sources of information than we could possibly cover. However, the nature of realist synthesis means that there is not a finite set of relevant papers to be found. Rather the reviewer takes a more purposive approach to sampling [129], with the aim of reaching conceptual saturation rather than identifying an exhaustive set of studies [21].

Many of the conclusions in this review about diabetes care are not specific to people with dementia. For example, personalised approaches to medication choice, continuity of care and a focus on individual patient preferences are relevant to all age groups [131, 132]. The review shows, however, how a diagnosis of dementia creates extra and different needs from those experienced by people with diabetes but without dementia. People with dementia and diabetes are more likely to be dependent on support from unpaid carers, may have more trouble accessing diabetes-related healthcare [11] and are at greater risk of complications such as hypoglycaemic episodes. Future research should consider the impact of involving family carers in SM interventions for people with diabetes and dementia, look at ways to improve medication management and explore how professionals can recognise when a person is no longer able to self-manage and provide appropriate support.

## Conclusions

The challenge for HCPs is how to accommodate quality of life, independence and patient and carer priorities with the minimum requirements of ‘good’ diabetic control. We recognise that perceptions of ‘good’ are situation-specific, differ for PLWDD and for family carers and will change over time. This review suggests that there is a need for a further work to establish a shared understanding of what needs to be in place to engage effectively with PLWD including those with diabetes and their supporters to establish how ‘good support’ is operationalised and measured. Much of the research included in this review, particularly that specific to PLWDD, identifies deficiencies and problems with current systems. Whilst we have highlighted the need for personalised care, continuity and family-centred approaches, there is much evidence to suggest that this is not currently happening. Future research on the management of diabetes in older people with complex health needs, including those with dementia, needs to look at how organisational structures and workforce development can be better aligned to their needs. The role of family carers in managing healthcare conditions of PLWD and their contribution in facilitating continuity and access to care are indisputable [11]. It is important, therefore, that HCPs conceptualise the provision of care for PLWD and a comorbidity as a complex phenomenon that affects not just individuals but also dyads and families [90].

## Box 1: Definitions of realist terms and how they have been applied in the review

- **Context (C):** This refers to the ‘backdrop’ conditions, for example provision of training in diabetes and/or dementia care delivery systems. Context can be broadly understood *as any condition that triggers and/or modifies the behaviour of a mechanism*
- **Mechanism (M):** A mechanism is the *generative force triggered in particular contexts* that leads to outcomes. It often denotes the reasoning (cognitive or emotional) of the various ‘actors’, i.e. people living with dementia and diabetes (*PLWDD*), relatives and healthcare professionals. Mechanisms are linked to, but are not the same as, a service’s strategies or interventions. Identifying the mechanisms goes *beyond describing* ‘what happened’ *to theorizing* ‘why it happened, for whom and under what circumstances’
- **Outcomes (O).** The outcome is a result of the interaction between a mechanism and its triggering context. Outcomes may include greater engagement in self-management behaviours or a reduction in adverse events
- **Programme theory:** Those ideas about what needs to be changed or improved in how diabetes is managed for PLWD, what needs to be in place to achieve improvement(s) and how programmes are believed to work. The programme theory specifies what is being investigated and the elements and scope of the review

## Abbreviations

AT: Assistive technology
CMO: Context-mechanism-outcome (configuration)
HCP: Healthcare professional
PAG: Project Advisory Group
PLWD: Person (people) living with dementia
PLWDD: Person (people) living with dementia and diabetes
RMT: Research Management Team
SM: Self-management
